# Altered lipid profile in uterine leiomyoma: a focus on apolipoprotein A1 reduction and machine learning-based predictive modeling

**DOI:** 10.3389/fmed.2026.1866505

**Published:** 2026-06-12

**Authors:** Feifei Zhang, Huizhen Lin, Huimin Yu, Yingsha Yao

**Affiliations:** Department of Gynecology, Ningbo No. 2 Hospital, Wenzhou Medical University, Ningbo, China

**Keywords:** apolipoprotein A1, machine learning, risk assessment, triglyceride, uterine leiomyomas

## Abstract

**Objective:**

This study aimed to investigate the association between uterine leiomyomas (UL) and specific alterations in serum lipid profiles, and to evaluate the performance of machine learning models incorporating these markers for UL discrimination.

**Methods:**

In this age- and body mass index matched case-control study, 200 patients with histologically confirmed UL and 200 controls with normal uteri were enrolled. Fasting serum levels of total cholesterol, triglycerides, low-density lipoprotein, high-density lipoprotein, apolipoprotein A1 (ApoA1), and apolipoprotein B were measured. Logistic regression identified independent risk factors, which were then used to construct predictive models via several machine learning algorithms. Model performance was assessed using receiver operating characteristic curve analysis.

**Results:**

Patients with UL exhibited significantly lower serum levels of triglycerides and ApoA1 compared to controls. Multivariate analysis confirmed lower triglyceride and ApoA1 levels, along with higher gravidity and premenopausal status, as independent factors associated with UL. While individual lipid parameters showed limited discriminative power, integrative models combining these with clinical features achieved high performance. The Random Forest model demonstrated superior discriminative ability, with an area under the curve of 0.986.

**Conclusion:**

After rigorous confounding control, UL is independently associated with a distinct metabolic phenotype characterized by reduced serum triglyceride and ApoA1 levels. Prediction models integrating these lipid abnormalities with clinical data show promising potential for risk assessment, highlighting a unique interplay between lipid metabolism and UL pathogenesis worthy of further investigation.

## 1 Introduction

Uterine leiomyomas (UL), recognized as the most prevalent non-cancerous tumors in the female reproductive system, pose a major health challenge for women worldwide. Research conducted in various nations reveals significant differences in the occurrence rates, with prevalence ranging from 4.5% to 68% and incidence rates between 2.2 and 37.5 cases per 1,000 person-years (1). This variability is closely linked to factors such as ethnicity (2), age (3), and hormonal status (4). Despite being classified as benign at the microscopic level, UL can cause a range of clinical problems, including irregular uterine bleeding, pelvic discomfort, pressure-related symptoms, and issues with reproduction. These symptoms can greatly diminish quality of life, limit everyday activities, and potentially lead to more extensive health complications, resulting in a considerable economic impact (5).

Dyslipidemia, a pathological state characterized by elevated serum levels of total cholesterol (TC), low-density lipoprotein cholesterol (LDL-C), and triglycerides (TG), and/or reduced high-density lipoprotein cholesterol (HDL-C), arises from an imbalance in lipid uptake, synthesis, transport, and clearance (6). Apolipoproteins (Apo), serving as the structural and functional core components of lipoproteins, play a pivotal role in these processes and are regulated by a combination of genetic, dietary, lifestyle, and endocrine factors. It is well-established that the development and progression of UL are closely linked to the regulation of sex hormones, particularly estrogen and progesterone (7, 8). Notably, estrogen exerts a well-defined modulatory influence on lipid metabolism, suggesting that the hormonal axis driving UL may concurrently interact with systemic lipid homeostasis. Supporting this notion, simvastatin–a lipid-lowering agent–has demonstrated anti-fibroid activity by modulating estrogen receptor alpha (ER-α) expression (9), further indicating a potential connection between abnormal lipid metabolism and leiomyoma growth. This interplay implies that the pathogenesis of UL may extend beyond local regulation within the reproductive endocrine system, involving broader and more complex interactions with systemic metabolic status.

Therefore, systematically elucidating the association between UL and dyslipidemia holds considerable scientific and clinical significance. From a scientific perspective, such research integrates the study of this common gynecological tumor into the broader framework of systemic metabolic disorders. Clinically, if specific lipid abnormalities are confirmed as independent correlates of UL, they could provide novel biomarkers for risk stratification and inform preventive strategies. Against this background, this case-control study aims to control for major confounding variables and systematically compare the baseline clinical characteristics and serum levels of TC, TG, LDL-C, HDL-C, and apolipoproteins between patients with UL and healthy controls. The findings are expected to provide higher-quality evidence on their epidemiological association and establish a foundation for subsequent mechanistic exploration and clinical translation.

## 2 Materials and methods

### 2.1 Study design and patient recruitment

This retrospective case-control investigation involved adult women who had gynecological procedures at our facility from November 2020 to October 2025. The group of cases included those diagnosed with uterine leiomyoma post-surgery, while the control group comprised individuals with a healthy uterus, verified through both surgical observation and subsequent pathology, who were treated for non-cancerous benign gynecological issues like pelvic organ prolapse or benign ovarian cysts. Participants were excluded based on the following criteria: (1) prior history of cancer or other systemic metabolic disorders (such as diabetes or thyroid issues); (2) recent use of drugs that could influence lipid metabolism (including statins or hormonal treatments) within 3 months before the operation; (3) being pregnant or breastfeeding; and (4) lack of essential clinical or laboratory information.

**FIGURE 1:**
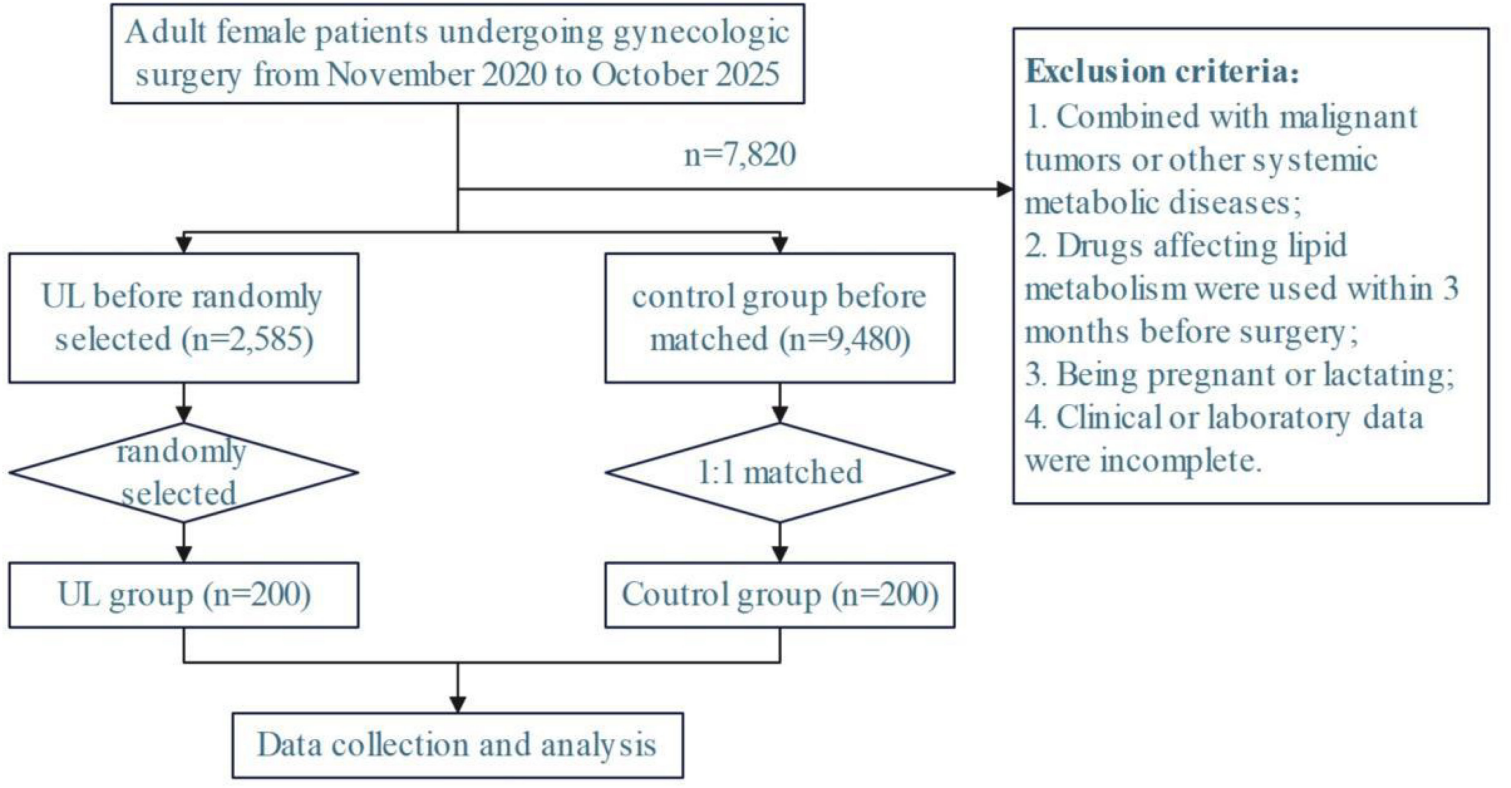
Flow of case data collection. UL, uterine leiomyoma.

Following the implementation of the specified criteria, a total of 200 patients diagnosed with uterine leiomyoma were randomly chosen for the case group utilizing the random sampling feature in Microsoft Excel 2019 (Microsoft Corporation, Redmond, WA, USA). A control group was created to match the case group in a 1:1 ratio, considering age and body mass index (BMI), leading to the inclusion of 200 controls. The process of selecting participants is illustrated in Figure 1.

### 2.2 Data collection

Baseline demographic characteristics, clinical history, and preoperative laboratory data were collected through the hospital’s electronic medical record system. Demographic data included age, BMI, obstetric history, and menopausal status. Fasting venous blood samples were collected from all patients within 1 week before surgery. Serum levels of TC, TG, LDL-C and HDL-C were measured using enzymatic methods on an automated biochemical analyzer (Cobas c702, Roche Diagnostics). ApoA1 and ApoB levels were determined by immunoturbidimetric assay. Lipoprotein(a) and other relevant indicators were also analyzed following standardized procedures. All laboratory tests were performed by the hospital’s central laboratory, with consistent methodologies and quality control maintained throughout the study period.

### 2.3 Ethical approval

The research adhered to the ethical guidelines outlined in the Declaration of Helsinki and received approval from the Ethics Committee at Ningbo No. 2 Hospital, Wenzhou Medical University (Approval No.: PJ-NBEY-KY-2025-279-01). Since this was a retrospective study utilizing completely anonymized clinical information that did not jeopardize patient privacy or interests, the Ethics Committee exempted the need for informed consent.

### 2.4 Statistical analysis

Data handling and verification through dual-entry were executed with Microsoft Excel 2019. For statistical evaluations, SPSS version 26.0 (IBM Corp., Armonk, NY, USA) and the SPSSPRO online platform (Version 1.0.11; Beijing QingSi Data Technology Co., Ltd.,) were utilized. Graphs were created using GraphPad Prism version 10.1.2 (GraphPad Software, San Diego, CA, USA).

The Shapiro–Wilk test was utilized to evaluate the normal distribution of continuous variables. Data that followed a normal distribution are reported as mean ± standard deviation (SD) and analyzed between groups through independent samples *t*-tests. For data that did not conform to a normal distribution, results are shown as median (interquartile range, IQR) and assessed using the Mann–Whitney U test. Categorical data are presented as counts (percentages) and examined with either the chi-square test or Fisher’s exact test, depending on the situation.

Initially, univariate logistic regression was conducted to assess the individual relationships between various factors and uterine leiomyoma. Those variables that showed a significance level of *p* < 0.05 in the univariate analysis, in addition to other clinically significant factors, were incorporated into a multivariable binary logistic regression model. This model utilized a forward stepwise selection method grounded in the likelihood ratio. The findings are presented as odds ratios (OR) accompanied by 95% confidence intervals (CI).

In order to evaluate the predictive capabilities, various machine learning algorithms–such as Back-Propagation Neural Networks, extremely randomized trees, random forests, and decision trees–were created utilizing the SPSSPRO software. The effectiveness of each risk factor and the various predictive models was analyzed by generating receiver operating characteristic (ROC) curves and determining the area under the curve (AUC). All analyses were conducted as two-tailed tests, with a significance threshold set at *p* < 0.05. The dataset was randomly divided into training and testing subsets at a ratio of 7:3, and the AUC values reported were all from the validation data.

## 3 Results

### 3.1 Baseline characteristics of the study population

A total of 200 patients with UL and 200 non-UL controls were included in this study. The participant selection flow is depicted in Figure 1. The two groups were well-matched in terms of age and BMI, with no statistically significant differences (both *p* > 0.05). However, significant inter-group differences were observed in gravidity, menopausal status, abnormal uterine bleeding (AUB), dysmenorrhea, and hypertension (all *p* < 0.05). Detailed baseline characteristics are presented in Table 1.

**TABLE 1:** Baseline characteristics of patients in the UL group and control group.

Characteristics	UL (*n* = 200)	Control (*n* = 200)	Statistical value	*P*-value
Age (years), mean ± SD	48.47 ± 7.16	47.06 ± 10.80	1.539	0.125
BMI (kg/m^2^), mean ± SD	23.49 ± 3.01	23.92 ± 3.06	−1.413	0.158
Gravidity, M (P25, P75)	2 (1, 3)	1 (1, 2)	8.701	<0.001
Parity, M (P25, P75)	1 (1, 1)	1 (1, 2)	−1.708	0.088
Menopausal status, *n* (%)	7 (3.5)	61 (30.5)	51.665	<0.001
AUB, *n* (%)	67 (33.5)	0 (0.0)	80.480	<0.001
Dysmenorrhea, *n* (%)	16 (8.0)	37 (18.5)	9.592	0.002
Hypertension, *n* (%)	10 (5.0)	0 (0.0)	10.256	0.001
Diabetes, *n* (%)	3 (1.5)	11 (5.5)	3.627	0.057

AUB, abnormal uterine bleeding; BMI, body mass index; UL, uterine leiomyoma.

### 3.2 Comparison of major lipid and apolipoprotein profiles

Compared with the control group, patients with UL exhibited significantly lower serum levels of TC, ApoA1, and lipoprotein(a) (all *p* < 0.05). The difference in ApoA1 levels was particularly notable (*p* < 0.001). Serum TG levels were also significantly lower in the UL group (*p* = 0.011). No significant differences were found in LDL-C, HDL-C, ApoB, or the ApoB/ApoA1 ratio between the groups (all *p* > 0.05). The comparative lipid profiles are detailed in Table 2.

**TABLE 2:** Main lipid and apolipoprotein profiles of patients with UL and controls.

Index	UL (*n* = 200)	Control (*n* = 200)	Statistical value	*P*-value
TC (mmol/L), mean ± SD	4.88 ± 0.95	4.90 ± 0.99	−0.207	0.836
TG (mmol/L), mean ± SD	1.33 ± 0.90	1.59 ± 1.14	−2.572	0.011
LDL-C (mmol/L), mean ± SD	2.84 ± 0.68	2.95 ± 2.16	−0.683	0.495
HDL-C (mmol/L), mean ± SD	1.45 ± 0.32	1.42 ± 0.33	0.981	0.327
ApoA1 (g/L), mean ± SD	1.37 ± 0.20	1.47 ± 0.22	−4.613	<0.001
TG/ApoA1 ratio, mean ± SD	1.00 ± 0.72	1.12 ± 0.87	−1.561	0.119
ApoB (g/L, mean ± SD)	0.80 ± 0.17	0.90 ± 0.67	−1.880	0.061
ApoB/ApoA1 ratio, mean ± SD	1.77 ± 0.40	1.81 ± 0.51	−0.879	0.380
ApoE (g/L), mean ± SD	6.42 ± 4.45	5.54 ± 1.24	9.592	0.002
Lipoprotein(a) (g/L), M (P25, P75)	70.85 (41.13, 146.03)	101.40 (56.10, 204.88)	−2.991	0.003

Apo, apolipoprotein; HDL-C, high-density lipoprotein cholesterol; LDL-C, low-density lipoprotein cholesterol; TC, total cholesterol; TG, triglycerides; UL, uterine leiomyoma.

### 3.3 Logistic regression analysis of risk factors for uterine leiomyoma

Logistic regression was performed on factors showing significant inter-group differences. AUB and dysmenorrhea were excluded from regression analysis due to the absence of positive cases in the control group. Univariate analysis revealed that higher gravidity, premenopausal status, absence of hypertension, lower TG levels, and lower ApoA1 levels were significantly associated with an increased risk of UL (all *p* < 0.05) (Table 3).

**TABLE 3:** Univariate logistic regression analysis of risk factors for uterine leiomyoma.

Variable	β	Wald χ^2^	OR	OR 95% CI	*P*-value
Gravidity	0.977	62.166	2.655	2.083–3.385	<0.001
Menopausal	−2.493	36.218	0.083	0.037–0.186	<0.001
Hypertension	−0.960	9.107	0.383	0.205–0.714	0.003
TG	−0.279	6.153	0.756	0.606–0.943	0.013
ApoA1	−2.250	19.240	0.105	0.039–0.288	<0.001
Lipoprotein(a)	−0.001	3.026	0.999	0.998–1.000	0.082

ApoA1, apolipoprotein A1; CI, confidence interval; OR, odds ratio; TG, triglycerides.

Variables with *p* < 0.05 in the univariate analysis were subsequently entered into a multivariate logistic regression model (forward stepwise method). The results identified higher gravidity (OR = 2.881, 95% CI: 2.198–3.775, *p* < 0.001), premenopausal status (OR = 0.097, 95% CI: 0.041–0.233, *p* < 0.001), lower TG levels (OR = 0.712, 95% CI: 0.544–0.933, *p* = 0.014), and lower ApoA1 levels (OR = 0.128, 95% CI: 0.039–0.424, *p* = 0.001) as independent factors associated with UL (Table 4). Notably, lower TG and ApoA1 levels were associated with an increased risk of UL in this model.

**TABLE 4:** Multivariate logistic regression analysis of risk factors for uterine leiomyoma.

Variable	β	Wald χ^2^	OR	OR 95% CI	*P*-value
Gravidity	1.058	58.837	2.881	2.198–3.775	<0.001
Menopausal	−2.331	27.331	0.097	0.041–0.233	<0.001
TG	−0.339	6.094	0.712	0.544–0.933	0.014
ApoA1	−2.054	11.333	0.128	0.039–0.424	0.001
Constant	1.960	4.529	7.096	–	0.033
Hypertension	–	–	–	–	0.336

ApoA1, apolipoprotein A1; CI, confidence interval; OR, odds ratio; TG, triglycerides.

### 3.4 Discriminative performance based on independent risk factors

A logistic regression discriminative model was constructed using the independent risk factors identified above (gravidity, menopausal status, TG, ApoA1). The optimal cut-off values for each key indicator were determined using the Youden index. Their diagnostic performance, including positive likelihood ratios, negative likelihood ratios, sensitivity, and specificity, is summarized in Table 5.

**TABLE 5:** Optimal cut-off values and diagnostic performance of key indicators.

Index	Cut-off value	Youden index	Positive likelihood ratios	Negative likelihood ratios	Sensitivity (%)	Specificity (%)
Gravidity	>1	0.3950	2.23	0.42	71.50	68.00
Menopausal	NO	0.2700	1.39	0.11	96.50	30.50
TG	≤1.59	0.2200	1.39	0.49	78.50	43.50
ApoA1	≤1.34	0.2200	1.81	0.70	49.00	73.00

ApoA1, apolipoprotein A1; TG, triglycerides.

**FIGURE 2:**
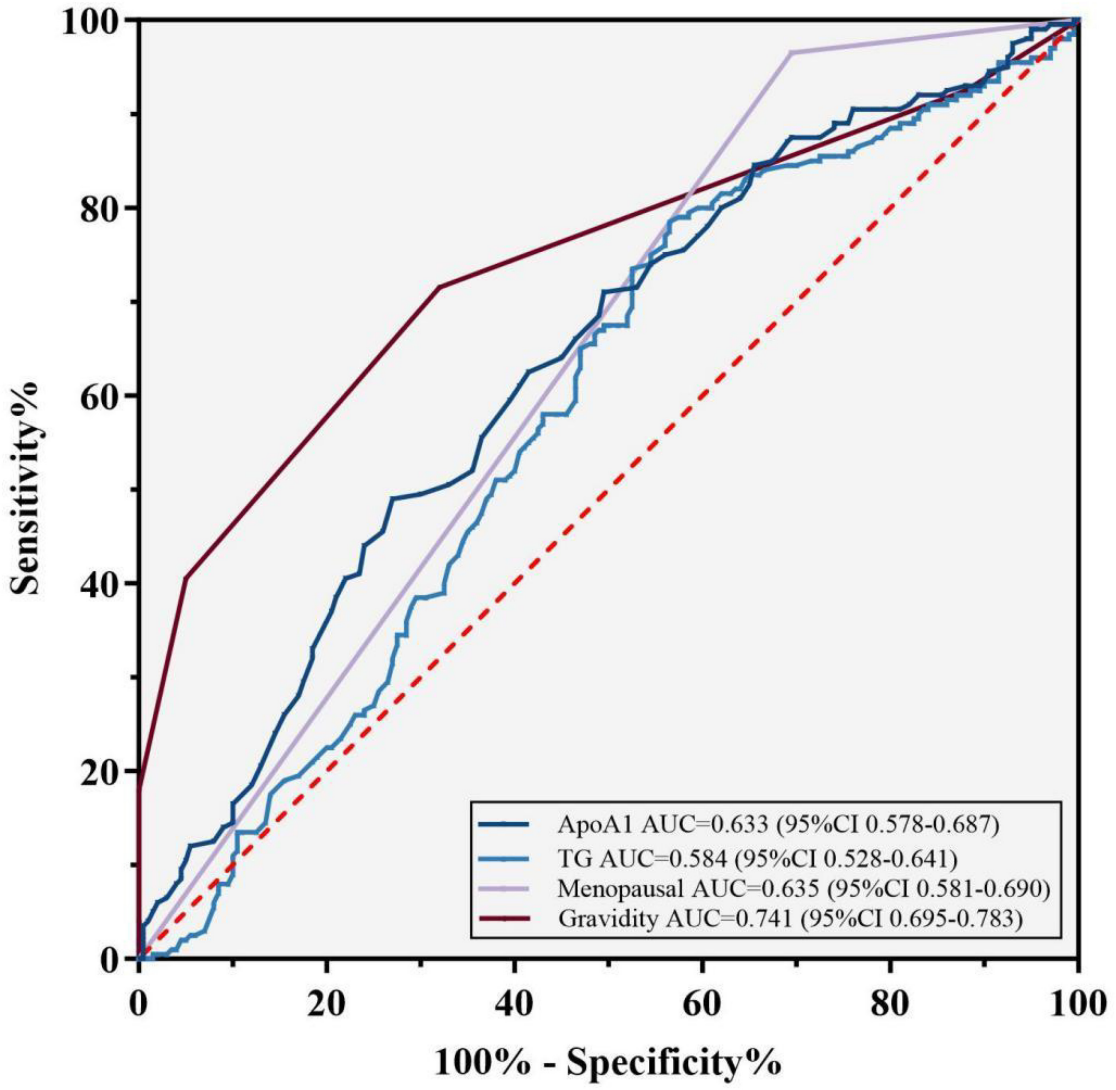
Receiver operating characteristic curve of single independent risk factor for discriminating uterine leiomyoma.

Receiver operating characteristic curve analysis (Figure 2) showed that the AUC for gravidity alone was 0.741 (95% CI: 0.695–0.783). The AUCs for menopausal status, TG, and ApoA1 were 0.635 (95% CI: 0.581–0.690), 0.584 (95% CI: 0.528–0.641), and 0.633 (95% CI: 0.578–0.687), respectively. Individually, these indicators showed either suboptimal sensitivity or specificity, limiting their standalone utility for discriminating UL.

### 3.5 Discriminative performance of various prediction models

The performance metrics of several machine learning models are presented in Table 6. For comparison, the conventional binary logistic regression model yielded a sensitivity of 59.50% and a specificity of 92.00%. Key evaluation metrics included accuracy (proportion of correctly classified samples), recall (sensitivity), precision (positive predictive value), and the F1-score (harmonic mean of precision and recall). In the random forest model, the feature importance rankings were gravidity 30.00%, TG 29.20%, ApoA1 28.50%, and menopause 12.30%. In the Extra Trees model, the rankings were gravidity 35.30%, ApoA1 23.80%, TG 21.70%, and menopause 19.20%.

**TABLE 6:** Evaluation results of machine learning models.

Model	Accuracy	Recall	Precision	F1-score	Sensitivity (%)	Specificity (%)
BPNN	0.740	0.740	0.741	0.740	61.50	90.00
Extra trees	0.885	0.885	0.886	0.885	95.50	87.50
Random Forest	0.945	0.945	0.945	0.945	98.50	91.50
Decision tree	0.888	0.888	0.891	0.887	89.00	88.50

BPNN, Back-Propagation Neural Network.

**FIGURE 3:**
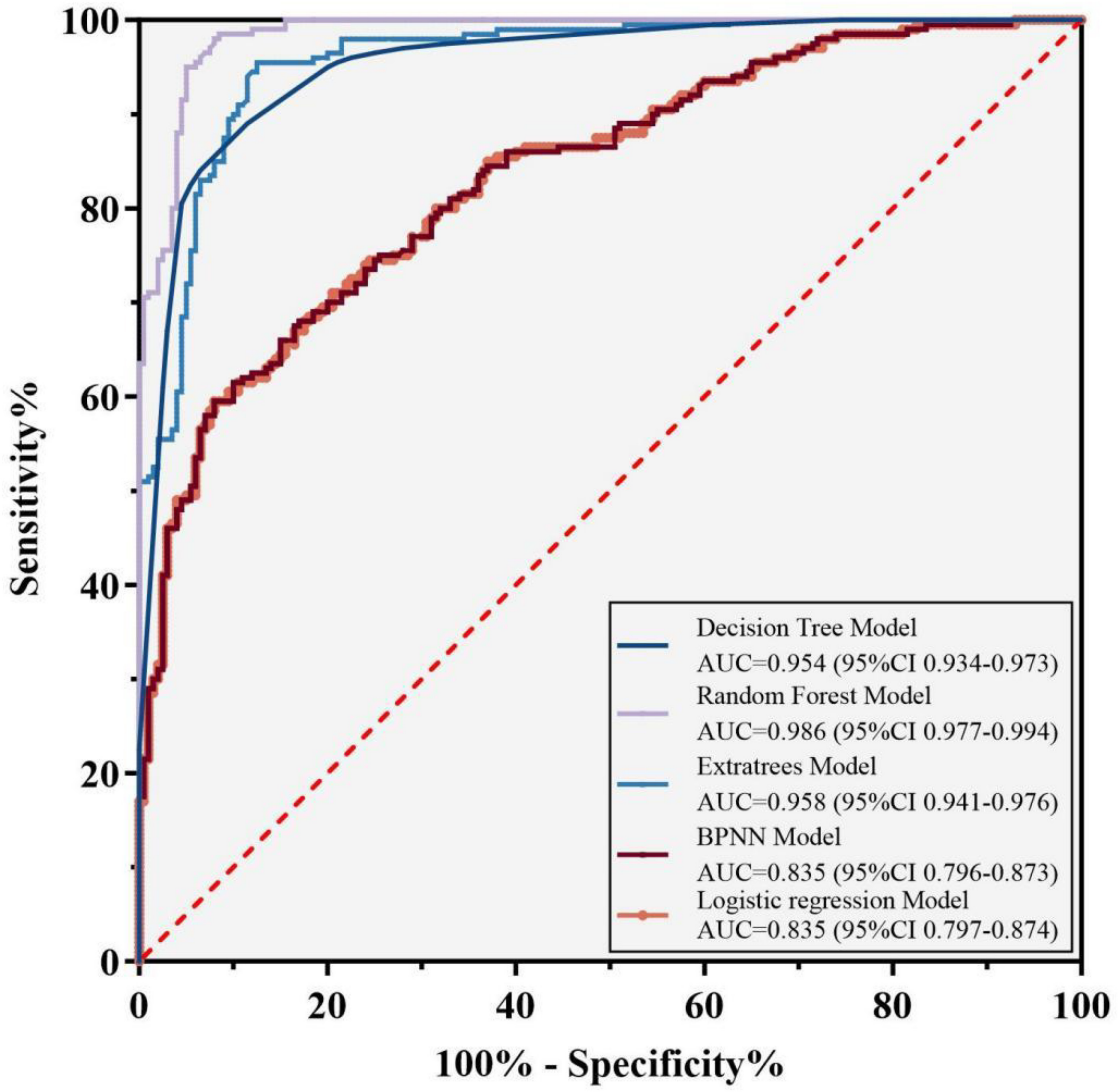
Receiver operating characteristic curve of multiple models for discriminating uterine leiomyoma (AUC, area under the receiver operating characteristic curve; BPNN, Back-Propagation Neural Network).

Receiver operating characteristic curve analysis (Figure 3) was used to further evaluate the models’ discriminative ability. The AUC for the logistic regression model was 0.835 (95% CI: 0.797–0.874). Among the machine learning models, the Back-Propagation Neural Network (BPNN) achieved an AUC of 0.835 (95% CI: 0.796–0.873), the Extra Trees model 0.958 (95% CI: 0.941–0.976), the Random Forest model 0.986 (95% CI: 0.977–0.994), and the Decision Tree model 0.954 (95% CI: 0.934–0.973). The ensemble methods, particularly Random Forest, demonstrated superior discriminative performance.

## 4 Discussion

This study, utilizing a case-control design with rigorous control for confounders, systematically compared the clinical characteristics and lipid metabolic profiles between patients with UL and control women. The primary finding was that lower levels of TG and ApoA1 were independently associated with UL. The results are discussed below in the context of existing literature.

### 4.1 Study population matching and confounding control

Key demographic and anthropometric measures, including age and BMI, were well-matched between the case and control groups.

This design effectively controlled for age and obesity, both established confounding factors significantly influencing both lipid profiles and UL risk (10–12). This strengthens the inference that the observed inter-group lipid differences are directly associated with UL itself, rather than merely reflecting these shared underlying factors. This finding underscores the critical importance of stringent control for major confounders when investigating relationships between metabolism and disease.

### 4.2 Association between UL and specific lipid-apolipoprotein profiles

Through rigorous case matching and multivariate regression, this study provides new clinical evidence supporting the hypothesis that UL is associated with specific lipoprotein metabolic abnormalities, particularly reduced ApoA1 levels, and offers a basis for further exploration of the potential implications of metabolic intervention in UL management.

Apolipoprotein A1, the primary functional protein of HDL-C, plays a central role in reverse cholesterol transport and is a key indicator of HDL-C function and cardiovascular protective capacity (13, 14). In contrast to the pro-atherogenic dyslipidemia features sometimes associated with UL in previous reports (15), the present study, after effectively controlling for key confounders like age and BMI, identified a metabolic alteration in UL patients characterized by significantly lower serum TG and ApoA1 levels. Multivariate analysis further confirmed the independent association of reduced ApoA1 with UL. Although prior literature has rarely directly reported reduced HDL-C or ApoA1 levels in UL patients, preclinical studies indicate that the cholesterol-lowering drug simvastatin inhibits leiomyoma cell proliferation by downregulating ER-α expression and transport, while simultaneously modulating estrogen signaling pathways (9). Although the ratio and effects of estrogen and progesterone receptors differ between fibroid cells and normal uterine smooth muscle cells, this suggests an interaction between cholesterol metabolism and estrogen regulatory networks.

### 4.3 Independent significance of lipid indicators and exploration of potential mechanisms

Multivariate logistic regression analysis identified higher gravidity, premenopausal status, lower TG levels, and lower ApoA1 levels as independent correlates of uterine leiomyoma. Notably, in this study, lower TG and ApoA1 levels were associated with a higher risk of UL.

This pattern contrasts with the classic cardiometabolic risk model, where elevated levels of these markers predict adverse outcomes (15), but aligns with observations in endometriosis research, where reduced ApoA1 and HDL-C levels have been linked to an increased risk of pelvic peritoneal endometriosis (16). This discrepancy suggests that the dyslipidemia associated with UL may involve a distinct pathophysiological mechanism. Consequently, we further explore potential biological intersections between UL and lipid metabolism abnormalities from the following perspectives.

First, the complex regulation of the sex hormone axis, particularly estrogen signaling, may be central to this association. Estrogen typically exerts a well-defined regulatory effect on lipid metabolism, with classical studies confirming its role in elevating HDL-C and its main functional protein, ApoA1 (17). However, this study found lower serum ApoA1 levels in UL patients, who were predominantly premenopausal. This paradoxical finding suggests the presence of specific aberrations in estrogen metabolic pathways, receptor responsiveness, or the balance between local and systemic hormonal action in these patients. For instance, altered transcriptional activity of ER-α or its downstream signaling pathways (e.g., ERK1/2) in leiomyoma tissue may weaken or even reverse the normal regulatory effect of estrogen on ApoA1 synthesis (18). Furthermore, environmental endocrine disruptors (e.g., bisphenol A, phthalates) may interfere with normal ER signaling function through epigenetic mechanisms (e.g., DNA methylation, miRNA expression regulation), thereby affecting ApoA1 expression and metabolism (19). This unique hormone-metabolism interaction warrants further in-depth investigation in future molecular and functional studies.

Second, chronic low-grade inflammation and associated metabolic remodeling may constitute a shared pathophysiological basis linking UL and lipid abnormalities. UL itself is considered associated with both local and systemic inflammatory states. Persistent low-grade inflammation may interfere with hepatic lipoprotein synthesis, secretion, and peripheral clearance, leading to characteristic alterations in the lipid profile. Existing literature indicates that chronic low-grade inflammation is a common pathological basis for various chronic diseases (e.g., sarcopenia, psoriasis), and triglyceride-rich lipoproteins themselves possess pro-inflammatory potential, which can further exacerbate systemic inflammation (20). Moreover, obesity and impaired glucose metabolism–both known drivers of inflammation–are also associated with UL risk and growth patterns (21), further supporting the potential involvement of inflammation in its pathogenesis. Notably, recent studies have found that low levels of ApoA1 are associated with an immunosuppressive state in the tumor microenvironment of cancers such as endometrial, ovarian, and lung cancer (22). As a key protein in reverse cholesterol transport, ApoA1 may restore macrophage function and activate anti-tumor immune responses through metabolic remodeling (e.g., by reducing levels of the oxysterol 7-ketocholesterol) (23). These mechanisms suggest that the lipid metabolism disorder pattern accompanying UL–particularly the reduction in ApoA1–may differ from classic inflammation-related metabolic abnormalities, and its specific mechanisms may be modulated by incompletely controlled confounding factors such as dietary patterns, genetic background, or body composition.

The metabolic features of uterine leiomyoma engage in complex bidirectional interactions with the local disease microenvironment. Research indicates that leiomyoma cells respond to hypoxia distinctly from normal myometrial cells, with hypoxia selectively driving leiomyoma growth, potentially via the HIF-1α pathway (24). Furthermore, fumarate hydratase-deficient leiomyomas exhibit activation of the NRF2 pathway (25), a key regulator of the oxidative stress response. This metabolic reprogramming may represent a compensatory response to the local microenvironment (e.g., hypoxia or inflammation), simultaneously promoting sustained tumor growth.

### 4.4 Discriminative performance of the models and preliminary clinical implications

Receiver operating characteristic curve analysis demonstrated that both the logistic regression model and various machine learning models (e.g., Random Forest) constructed based on the aforementioned independent risk factors exhibited good discriminative performance for UL, with the Random Forest model achieving the highest area under the curve (AUC) of 0.986. Although the discriminative ability of individual lipid indicators (e.g., TG, ApoA1) was limited, integrating them with clinical features (gravidity, menopausal status) significantly enhanced model performance. This suggests that comprehensive prediction models combining clinical information with specific lipid indicators may aid in identifying high-risk populations for UL in the future. However, their clinical application potential requires further validation in prospective cohort studies.

It is noteworthy that previous research has primarily applied machine learning models to the classification and diagnosis of uterine leiomyoma subtypes based on imaging data (26), whereas this study shifts the focus of modeling to the combined analysis of clinical indicators and lipid metabolism parameters, offering a new approach for auxiliary risk assessment.

### 4.5 Limitations

This study has several limitations. First, the cross-sectional design precludes causal inference regarding the relationship between changes in TG&ApoA1 levels and UL. Second, the single-center design and limited sample size restrict the generalizability of the findings; moreover, subgroup analyses for hypertension and other lipid-related factors could not be meaningfully conducted given the limited sample size, which may have constrained the assessment of effect modification. Third, although major confounders were controlled, residual confounding may exist due to unmeasured variables such as detailed dietary habits, physical activity levels, precise insulin resistance indices, and sex hormone levels. Finally, the study focused on serological associations using exploratory machine learning approaches and lacks direct mechanistic evidence at the tissue or cellular level. Therefore, the results should be interpreted as hypothesis-generating rather than confirmatory. Furthermore, the machine learning models, while demonstrating high performance internally, were developed without rigorous cross-validation or detailed hyperparameter optimization and are inherently exploratory. Their reported metrics, especially the high AUC for the Random Forest model, should be interpreted with caution as they may be optimistic due to overfitting. Therefore, the results should be interpreted as hypothesis-generating rather than confirmatory. Additionally, these models require external validation in prospective, multi-center cohorts before any clinical application can be considered.

## 5 Conclusion

In summary, this study, with careful control for confounding factors, found that uterine leiomyoma is independently associated with lower serum levels of TG and ApoA1–a metabolic phenotype distinct from the common pro-atherogenic pattern. It highlights the importance of strictly controlling for confounders like menopausal status when investigating metabolic associations in UL. Future research should involve large-scale prospective studies to clarify whether these lipid characteristics are risk markers or consequences of UL. Furthermore, basic experimental studies are needed to explore the underlying biological mechanisms, particularly the unique pathways involving the interplay between sex hormone metabolism, local inflammation, and systemic lipid homeostasis.

## Data Availability

The raw data supporting the conclusions of this article will be made available by the authors, without undue reservation.

## References

[1] Wilson LF Moss KM Doust J Farquhar CM Mishra GD. First Australian estimates of incidence and prevalence of uterine fibroids: a data linkage cohort study 2000–2022. *Hum Reprod.* (2024) 39:2134–43. 10.1093/humrep/deae162 39013145 PMC11373412

[2] Li Y McNally RP Feng Y Kim JJ Wei JJ. Racial differences in transcriptomics and reactive oxygen species burden in myometrium and leiomyoma. *Hum Reprod.* (2023) 38:609–20. 10.1093/humrep/dead020 36749068 PMC10068273

[3] Wegienka G Havstad S Coleman C Cooper T Wesselink A Upson K et al. Ultrasound-confirmed, age-specific uterine leiomyoma incidence in a cohort of black individuals. *Obstet Gynecol.* (2022) 140:1042–8. 10.1097/AOG.0000000000004997 36357982 PMC9712257

[4] Yang Q Ciebiera M Bariani MV Ali M Elkafas H Boyer TG et al. Comprehensive review of uterine fibroids: developmental origin, pathogenesis, and treatment. *Endocr Rev.* (2022) 43:678–719. 10.1210/endrev/bnab039 34741454 PMC9277653

[5] Dai Y Chen H Yu J Cai J Lu B Dai M et al. Global and regional trends in the incidence and prevalence of uterine fibroids and attributable risk factors at the national level from 2010 to 2019: a worldwide database study. *Chin Med J (Engl)*. (2024) 137:2583–9. 10.1097/CM9.0000000000002971 38407293 PMC11556989

[6] Pirillo A Casula M Olmastroni E Norata GD Catapano AL. Global epidemiology of dyslipidaemias. *Nat Rev Cardiol.* (2021) 18:689–700. 10.1038/s41569-021-00541-4 33833450

[7] Cloud AS Koohestani F McWilliams MM Ganeshkumar S Gunewardena S Graham A et al. Loss of the repressor REST affects progesterone receptor function and promotes uterine leiomyoma pathogenesis. *Proc Natl Acad Sci U S A.* (2022) 119:e2205524119. 10.1073/pnas.2205524119 36282915 PMC9636955

[8] Milewska G Ponikwicka-Tyszko D Bernaczyk P Lupu O Szamatowicz M Sztachelska M et al. Functional evidence for two distinct mechanisms of action of progesterone and selective progesterone receptor modulator on uterine leiomyomas. *Fertil Steril.* (2024) 122:341–51. 10.1016/j.fertnstert.2024.02.046 38431184

[9] Afrin S El Sabeh M Miyashita-Ishiwata M Charewycz N Singh B Borahay MA. Simvastatin reduces plasma membrane caveolae and caveolin-1 in uterine leiomyomas. *Life Sci.* (2022) 304:120708. 10.1016/j.lfs.2022.120708 35705139

[10] De Bruyn C Ceusters J Vanden Brande K Timmerman S Froyman W Timmerman D et al. Ultrasound features using MUSA terms and definitions in uterine sarcoma and leiomyoma: cohort study. *Ultrasound Obstet Gynecol.* (2024) 63:683–90. 10.1002/uog.27535 37970762

[11] Sharma R Diwan B. Lipids and the hallmarks of ageing: from pathology to interventions. *Mech Ageing Dev.* (2023) 215:111858. 10.1016/j.mad.2023.111858 37652278

[12] Saad EE Michel R Borahay MA. Senescence-associated secretory phenotype (SASP) and uterine fibroids: association with PD-L1 activation and collagen deposition. *Ageing Res Rev.* (2024) 97:102314. 10.1016/j.arr.2024.10231 38670462 PMC11181954

[13] Bhale AS Venkataraman K. Leveraging knowledge of HDLs major protein ApoA1: structure, function, mutations, and potential therapeutics. *Biomed Pharmacother.* (2022) 154:113634. 10.1016/j.biopha.2022.113634 36063649

[14] Laguna-Maldonado KD Uribe-Ramírez D Vázquez-Carrada M Matuz-Mares D Vilchis-Landeros MM. Dysfunction of the ABCA1 and ABCG1 transporters and their impact on HDL metabolism. *Antioxidants (Basel)*. (2025) 14:1362. 10.3390/antiox14111362 41300518 PMC12649166

[15] Schaefer EJ Asztalos BF Vaisar T Diffenderfer MR Brewer HB Stock EO et al. High density lipoprotein particle composition, functionality, deficiency, and atherosclerotic cardiovascular disease risk: a review. *Curr Atheroscler Rep*. (2025) 27:62. 10.1007/s11883-025-01308-9 40489011

[16] He X Xie S Liu Y. The association of circulating lipoprotein lipids and apolipoproteins with risk of endometriosis: a Mendelian randomization study. *Postgrad Med J.* (2024) 100:578–83. 10.1093/postmj/qgae011 38491971

[17] Afrin S El Sabeh M Islam MS Miyashita-Ishiwata M Malik M Catherino WH et al. Simvastatin modulates estrogen signaling in uterine leiomyoma via regulating receptor palmitoylation, trafficking and degradation. *Pharmacol Res.* (2021) 172:105856. 10.1016/j.phrs.2021.105856 34461224 PMC8455458

[18] Wang J Xu P Zou G Che X Jiang X Liu Y et al. Integrating spatial transcriptomics and single-nucleus RNA sequencing reveals the potential therapeutic strategies for uterine leiomyoma. *Int J Biol Sci.* (2023) 19:2515–30. 10.7150/ijbs.83510 37215998 PMC10197899

[19] Mlodawska OW Saini P Parker JB Wei JJ Bulun SE Simon MA et al. Epigenomic and enhancer dysregulation in uterine leiomyomas. *Hum Reprod Update.* (2022) 28:518–47. 10.1093/humupd/dmac008 35199155 PMC9247409

[20] Kamper RS Alcazar J Andersen LL Haddock B Jørgensen NR Hovind P et al. Associations between inflammatory markers, body composition, and physical function: the Copenhagen Sarcopenia Study. *J Cachexia Sarcopenia Muscle.* (2021) 12:1641–52. 10.1002/jcsm.12832 34708570 PMC8718077

[21] Manuel EC Plowden TC Valbuena FM Bryce RL Barick AA Ramakrishnan A et al. The environment, leiomyomas, latinas, and adiposity study: rationale and design. *Am J Obstet Gynecol.* (2022) 226:.392.e1–12. 10.1016/j.ajog.2021.05.005 33974903

[22] Lv Q Su T Liu W Wang L Hu J Cheng Y et al. Low serum apolipoprotein A1 levels impair antitumor immunity of CD8+ T cells via the HIF-1α -glycolysis pathway. *Cancer Immunol Res.* (2024) 12:1058–73. 10.1158/2326-6066.CIR-23-0506 38752667

[23] Wang S Yan W Kong L Zuo S Wu J Zhu C et al. Oncolytic viruses engineered to enforce cholesterol efflux restore tumor-associated macrophage phagocytosis and anti-tumor immunity in glioblastoma. *Nat Commun.* (2023) 14:4367. 10.1038/s41467-023-39683-z 37474548 PMC10359270

[24] Miyashita-Ishiwata M El Sabeh M Reschke LD Afrin S Borahay MA. Differential response to hypoxia in leiomyoma and myometrial cells. *Life Sci.* (2022) 290:120238. 10.1016/j.lfs.2021.120238 34942165 PMC8757389

[25] Mehine M Ahvenainen T Khamaiseh S Härkönen J Reinikka S Heikkinen T et al. A novel uterine leiomyoma subtype exhibits NRF2 activation and mutations in genes associated with neddylation of the Cullin 3-RING E3 ligase. *Oncogenesis*. (2022) 11:52. 10.1038/s41389-022-00425-3 36068196 PMC9448808

[26] Tamehisa T Sato S Sakai T Maekawa R Tanabe M Ito K et al. Establishment of noninvasive prediction models for the diagnosis of uterine leiomyoma subtypes. *Obstet Gynecol.* (2024) 143:358–65. 10.1097/AOG.0000000000005475 38061038

